# Chemical, Thermal and Spectroscopic Methods to Assess Biodegradation of Winery-Distillery Wastes during Composting

**DOI:** 10.1371/journal.pone.0138925

**Published:** 2015-09-29

**Authors:** A. Torres-Climent, P. Gomis, J. Martín-Mata, M. A. Bustamante, F. C. Marhuenda-Egea, M. D. Pérez-Murcia, A. Pérez-Espinosa, C. Paredes, R. Moral

**Affiliations:** 1 Department of Agrochemistry and Environment, Miguel Hernandez University, Orihuela, Alicante, Spain; 2 Department of Agrochemistry and Biochemistry, University of Alicante, Alicante, Spain; INSTITUTO MEXICANO DEL PETRÓLEO, MEXICO

## Abstract

The objective of this work was to study the co-composting process of wastes from the winery and distillery industry with animal manures, using the classical chemical methods traditionally used in composting studies together with advanced instrumental methods (thermal analysis, FT-IR and CPMAS ^13^C NMR techniques), to evaluate the development of the process and the quality of the end-products obtained. For this, three piles were elaborated by the turning composting system, using as raw materials winery-distillery wastes (grape marc and exhausted grape marc) and animal manures (cattle manure and poultry manure). The classical analytical methods showed a suitable development of the process in all the piles, but these techniques were ineffective to study the humification process during the composting of this type of materials. However, their combination with the advanced instrumental techniques clearly provided more information regarding the turnover of the organic matter pools during the composting process of these materials. Thermal analysis allowed to estimate the degradability of the remaining material and to assess qualitatively the rate of OM stabilization and recalcitrant C in the compost samples, based on the energy required to achieve the same mass losses. FT-IR spectra mainly showed variations between piles and time of sampling in the bands associated to complex organic compounds (mainly at 1420 and 1540 cm^-1^) and to nitrate and inorganic components (at 875 and 1384 cm^-1^, respectively), indicating composted material stability and maturity; while CPMAS ^13^C NMR provided semi-quantitatively partition of C compounds and structures during the process, being especially interesting their variation to evaluate the biotransformation of each C pool, especially in the comparison of recalcitrant C vs labile C pools, such as Alkyl /O-Alkyl ratio.

## Introduction

Currently, the European Union (EU-27) is the world’s leader in the wine sector, with 55% of the global vineyard and around 60% of wine production, France, Italy and Spain being the largest world wine producing countries [1], which implies a great generation of wastes, whose disposal and recycling constitute an environmental problem. Grape marc (GM) and exhausted grape marc (EGM) constitute some of the main solid wastes generated. According to the Council Regulation (EC) No 491/2009 on the common organization of agricultural markets and on specific provisions for certain agricultural products, GM, the primary waste of wine production obtained after the pressing process, is considered a by-product and must be sent to alcohol distilleries to extract alcohol and tartrates, producing a solid waste, exhausted grape marc (EGM), obtained after the grape marc-washing processes [2].

Several alternatives have been proposed to recycle these wastes and thus, minimize the potential environmental risk associated to their disposal, which include simple options, such as the use for feedstuff production, and advanced treatments to obtain added-value products (grape seed oil, polyphenolic compounds) or energy [2,3]. However, the obtaining of different substances from these wastes depends on the market value of the obtained products related to the management of the waste from the winemaking process. Moreover, in most of the previous treatments (except the production of feedstuff or energy), the amount of remained waste is close to that prior to be treated.

Composting is a widely used technology to transform residual organic matter, with a significant reduction of volume and weight during the process, in a sanitized, stabilized and mature product, easy to store and marketable as organic amendment or fertilizer [4]. Therefore, the composting of the winery-distillery wastes can constitute a feasible option not only to manage these wastes, but also to recycle them, as it has been reported by different authors [5, 6]. However, more studies have been carried about the co-composting of the winery-distillery wastes with other organic residues, such as agroindustrial wastes [7], animal manures [8, 9] and urban wastes [9, 10]. Although these authors have reported the composting of winery wastes, enough attention has not been put onto organic matter changes and the composting process itself. In this complex biological aerobic system, it is essential to obtain an in-depth understanding of the organic matter transformations, in order to optimize the composting process and the later application of the compost obtained [11]. This is especially important when specific waste fluxes are considered, such as the winery-distillery wastes and their potential synergies with manures as co-composting ingredients.

Currently, there is a clear consensus about the absence of one specific parameter to ascertain stability, maturity and /or quality in compost using an only technique, due to the high variability observed in the composts, since their characteristics strongly depend on the raw material and the composting system used [12]. This fact is especially notable in certain materials, such as the winery-distillery wastes. These materials usually show an anomalous trend of the humification indices (usual parameters considered to evaluate the process development) during composting, when the classical and non-specific analytical methods are used [8]. Therefore, one of the best solutions to avoid this could be the knowledge of the overall process and of the evolution of organic matter [13]. So, the combination of classical techniques and advanced instrumental methods can provide more conclusive information about the process [14]. In this sense, the thermal and spectroscopic techniques show some advantages compared to the classical analytical methods, usually time-consuming and that require complex sample preparations and/or specific reactants [15].

The instrumental techniques used in this work (thermal analysis, FT-IR and CPMAS ^13^C NMR techniques) were selected due to their efficiency for the characterization of the organic matter pools in different organic materials, especially composts. Thermal decomposition analyses, including thermogravimetry (TG), derivative thermogravimetry (DTG) and differential thermal analysis (DTA) have been reported as useful tools for structural and chemical assessment of natural organic matter [16, 17, 18,19]. Comparative mass loss analysis between samples during composting can inform about the changes into the complexity of organic matter structures [16]. Moreover, correlation of DTG profiles and time variation in samples can help to identify specific types and organic matter transformations [17, 19].

Fourier transform infrared spectroscopy (FT-IR) constitutes an useful technique to study the development of the composting process, since it can help in the qualitative characterization of the principal chemical groups of the raw organic matter [20, 21, 22]. With FT-IR spectra, the relative intensity of the typical bands of components can be selected and used to follow the composting process, monitoring the transformation of the organic and inorganic materials.

Solid-state ^13^C magnetic resonance spectroscopy (solid-state C^13^ NMR) constitutes one of the most powerful tools for studying the carbon composition of organic matter[21], since this technique allows to investigate samples without any need of extraction and fractionation and thus, to collect direct information on the structural characteristic of whole organic matter during the composting process [23]. So, several studies have proposed the cross-polarization and magic angle spinning (CPMAS) ^13^C NMR technique for monitoring the stabilization process during composting, analyzing the complete sample and the extracted humic acids [13, 19, 23].

Therefore, the main aim of this work was to study the development of the co-composting process of winery-distillery wastes with animal manures using the traditional analytical methods together with advanced instrumental methods (thermal analysis, FT-IR and CPMAS ^13^C NMR techniques) to ascertain organic matter changes during the process. The quality assessment of the final composts obtained was also carried out.

## Materials and Methods

### Compost procedure

Three different piles (A, B and C) were prepared by mixing wastes from the winery and distillery industry (grape marc (GM) and exhausted grape marc (EGM)), with two different animal manures (cattle manure (CM) and poultry manure (PM)). GM was obtained from a winery placed in Bullas (Murcia, Spain), EGM was collected from an alcohol distillery placed in Villarrobledo (Albacete, Spain); CM was obtained from a cattle farm in Santomera (Murcia, Spain) and PM was collected from a poultry farm of laying hens located in Orihuela (Alicante, Spain). The main characteristics of the raw materials are shown in Table 1.

**Table 1:** Main physico-chemical and chemical characteristics of the raw materials used in the composting piles (dry matter basis).

	EGM	GM	CM	PM
pH	6.22 ± 0.01	4.36 ± 0.01	9.92 ± 0.02	7.50 ± 0.01
EC (dS m^-1^)	1.46 ± 0.04	3.32 ± 0.02	7.28 ± 0.01	6.75 ± 0.06
OM (%)	91.9 ± 0.1	92.4 ± 0.0	70.3 ± 0.2	65.2 ± 0.5
TOC (%)	49.3 ± 0.35	50.9 ± 0.14	32.7 ± 0.21	31.4 ± 0.4
TN (%)	2.04 ± 0.01	1.78 ± 0.04	3.36 ± 0.01	4.57 ± 0.01
C/N ratio	24.2 ± 0.1	28.6 ± 1.1	9.73 ± 0.15	6.87 ± 0.17
WSC (%)	3.22 ± 0.02	4.07 ± 0.05	3.95 ± 0.01	4.74 ± 0.04
WSPOL (mg kg^-1^)	536 ± 5	1041 ± 12	3521 ± 23	8360 ± 40
P (g kg^-1^)	1.45 ± 0.20	1.03 ± 0.01	8.92 ± 0.32	9.60 ± 0.18
K (g kg^-1^)	6.1 ± 0.1	19.3 ± 0.1	27.4 ± 0.3	28.5 ± 0.1
Ca (g kg^-1^)	15.9 ± 0.2	10.9 ± 0.4	96.0 ± 0.7	104 ± 1.1
Mg (g kg^-1^)	1.20 ± 0.03	1.60 ± 0.02	15.4 ± 0.02	7.54 ± 0.06
Na (g kg^-1^)	0.27 ± 0.01	0.28 ± 0.00	16.3 ± 0.01	4.07 ± 0.02
Fe (mg kg^-1^)	624 ± 3	719 ± 9	1600 ± 14	171 ± 5
Mn (mg kg^-1^)	20.2 ± 0.8	17.2 ± 1.0	329 ± 11	279 ± 6
Cu (mg kg^-1^)	16.9 ± 0.1	19.0 ± 0.1	44.0 ± 0.2	47.0 ± 0.4
Zn (mg kg^-1^)	17.7 ± 0.2	18.3 ± 0.1	362 ± 3	264 ± 7

EGM: exhausted grape marc; GM: grape marc; CM: cattle manure; PM: poultry manure. EC: electrical conductivity; OM: organic matter; TOC: total organic carbon; TN: total nitrogen; WSC: water-soluble C; WSPOL: water-soluble polyphenols. Values reported as mean ± standard error (n = 3).

Then, the raw materials were thoroughly mixed, and each mixture obtained (about 150 kg) was separately placed in thermo-composters with and efficient volume of 350 L. The thermocomposters, 70 cm x 70 cm x 85 cm, were made of high-density polyethylene (HDPE) and have a lateral system of natural ventilation to guarantee aerobic conditions. The moisture of the piles was controlled weekly by adding the necessary amount of water to obtain a moisture content not less than 40%.

In order to compost the maximum weight of winery-distillery wastes with a sufficient amount of animal waste, as nitrogen and micro-organisms source, the composting mixtures were prepared on a dry weight basis (fresh weight basis in brackets) in the following proportions:

Pile A:76%EGM+24%CM[70:30];C/N ratio=21.9

Pile B:72%GM+28%CM[70:30];C/N ratio=21.1

Pile C:67%EGM+33%PM[70:30];C/N ratio=14.6

The mixtures were composted in the thermo-composters by the turning composting system. The piles were turned six times, when the temperature in the mixtures decreased, to provide aeration. The bio-oxidative phase of composting was considered finished when the temperature was close to the ambient and re-heating did not occur. Then, composts were left to mature over a period of two months, approximately.

Samples were taken at seven sites of the pile from the whole profile (from the top to bottom). Composite representative samples were obtained after mixing and homogenizing thoroughly the previous seven sub-samples.

Each sample was divided into two fractions: one of them was dried in a drying-oven at 105°C for 24 h to determine the moisture content and the second was air-dried and ground to less than 0.5 mm for the rest of the classical analytical determinations. For the thermal and spectroscopic analyses, composting samples were air-dried, ground in an agate mill, then sieved through a 0.125 mm mesh, and milled again with an agate mortar.

### Classical determinations: chemical analyses

In the raw materials and in the composting samples, EC and pH were analyzed in a 1:10 (w/v) water-soluble extract. Organic matter (OM) was assessed by determining the loss-on ignition at 500°C for 24 h; water-soluble organic carbon (WSC) was determined in a 1:10 (w/v) water extract by using an automatic carbon analyzer for liquid samples (TOC-V CSN Analyzer, Shimadzu).

Total organic C (TOC) and total N (TN) were determined were determined by dry combustion at 950°C using a Leco TruSpec C–N Elemental Analyzer (Leco Corp., St. Joseph, MI, USA). The humic-like fractions (extractable organic carbon (Cext), fulvic acid-like carbon (Cfa), and humic acid-like carbon (Cha)) were also determined using an automatic carbon analyzer for liquid samples (TOC-V CSN Analyzer, Shimadzu); these extractions were carried out according to the methods used by Bustamante et al. [8]. After HNO_3_/HClO_4_ digestion, P was assessed colorimetrically as molybdovanadate phosphoric acid, Na and K were determined by flame photometry (Jenway PFP7 Flame Photometer, Jenway Ltd., Felsted, UK) and Ca, Mg, Fe, Cu, Mn, Zn by inductively coupled plasma-optical emission spectrometry (ICP-OES, Thermo Elemental Co. Iris Intrepid II XDL, USA). All the analyses were made in triplicate. The germination index (GI) was calculated using seeds of *Lepidium sativum* L. according to the method of Zucconi et al. [24]. The humification indexes (HR, HI, Pha, Cha/Cfa) and the losses of OM (from the initial (X_1_) and final (X_2_) ash contents) were calculated according to the equations described by Bustamante et al. [8].

$$Humification\;ratio\;\left(HR\right)=100\;\left(\frac{Cext}{C_{T}}\right)$$

$$Humification\;index\;\left(HI\right)=100\;\left(\frac{Cha}{C_{T}}\right)$$

$$Percentage\;of\;humic\;acids\;\left(Pha\right)=100\;\left(\frac{Cha}{Cext}\right)$$

$$Polymerisation\;rate=\frac{Cha}{Cfa}$$

$$OM\;loss\;(\%)=100-100\;\frac{\left[X_{1}\;\left(100-X_{2}\right)\right]}{\left[X_{2}\;\left(100-X_{1}\right)\right]}$$

### Advanced instrumental determinations: thermal and spectroscopic analyses

Thermal analyses were performed with a Mettler Toledo (TGA/SDTA851e/LF/1600) and Pfeiffer Vacuum (Thermostar GSD301T) mass spectrometer that enables the recording of thermograms and mass spectra of combustion gases simultaneously. All samples were combusted with a mixing stream of oxygen/He (20/80%), a gas flow 100 ml min^−1^ within a temperature range from 25 to 650°C, a heating rate 10°C min^−1^, a sample weight around 5 mg, Al_2_O_3_ pan, and self-controlled calibration.

The FT-IR spectra were collected on a Bruker IFS 66 spectrometer. The resolution was set to 4 cm^−1,^ and the operating range was 400−4000 cm^−1^. The analytical technic used was FT-IR attenuated total reflection spectroscopy (ATR). Samples (7−10 mg) were mixed with 100 mg of dried KBr, and then the mixture was pressed into pellets. In all cases, 20 scans per sample were recorded, averaged for each spectrum, and corrected against the spectrum with ambient air as background.

CPMAS ^13^C NMR experiments were performed on a Bruker Advance DRX500 operating at 125.75 MHz for ^13^C. Samples were packed into a 4 mm diameter cylindrical zirconia rotor with Kel-F end-caps and spun at 10000 ± 100 Hz. A conventional CPMAS pulse sequence [25] was used with a 1.0 ms contact time. Between 2000 and 5000 scans were accumulated with a pulse delay of 1.5 s. Line broadening was adjusted to 50 Hz. Spectral distributions (the distribution of total signal intensity among various chemical shift ranges) were calculated by integrating the signal intensity in seven chemical shift regions: carbonyl (210−165 ppm), O-aromatic (165−145 ppm), aromatic (145−110 ppm), O_2_-alkyl (110−95 ppm), O-alkyl (95−60 ppm), N-alkyl/methoxy (60−45 ppm), and alkyl (45 to −10 ppm) [26]. The labels only indicate major types of C found in each region. Spin counting calculation were performed using the method of Smernik and Oades [27]. Glycine (analytical reagent grade; SIGMA) was used as an external intensity standard. The proton spin-lattice relaxation time (*T*_1_H) and the proton spin-lattice relaxation rate in the rotating frame (*T*_1ρ_ H) were determined as described in Smernik and Oades [27]. These parameters are very important in order to avoid signal loss in the CP-1 MAS spectra, since it is possible to choose the best conditions of the CP-MAS pulse sequence (these experimental conditions have been previously described). The percentage of potential ^13^C NMR signal, which was actually observed (Cobs), was in the range 60–66% for the CP-MAS technique [27]. The main source of error was uncertainty in the integrated NMR intensities. Two replicate measurements were carried out for all samples.

### Statistical analysis

Data corresponding to OM losses (OM degradation) produced throughout the composting process were fitted to a kinetic function by the Marquardt-Levenberg algorithm to minimize the sum of the squared differences between the observed and predicted values of the dependent variable, using the Sigmaplot 11.0 computer program. A first-order kinetic model was used for OM degradation during composting [28]. This model was chosen as the best fit because it gave a randomized distribution of the residuals together with the lowest residual mean square (RMS) value and a highly-significant F-value (data not shown). The model was:

$$\text{OM losses}\;\left(\%\right)=\text{A}\;\left(1-{\text{e}}^{-k\text{t}}\right);$$

where A is the maximum degradation of OM (% C_T_), *k* the rate constant (d^-1^) and t the composting time (d). The RMS and F-values were calculated to compare the fittings of different functions and the statistical significance of curve fitting. MATLAB version 6.5 from MathWorks was used for the calculations, as well as the iToolbox (including methods for iPLS, biPLS and dynamic biPLS), EMSC Toolbox (for pre-processing methods), and GA-PLS Toolbox, available from http://www.models.kvl.dk.

## Results and Discussion

### Temperature evolution of the composting piles

**Fig 1:**
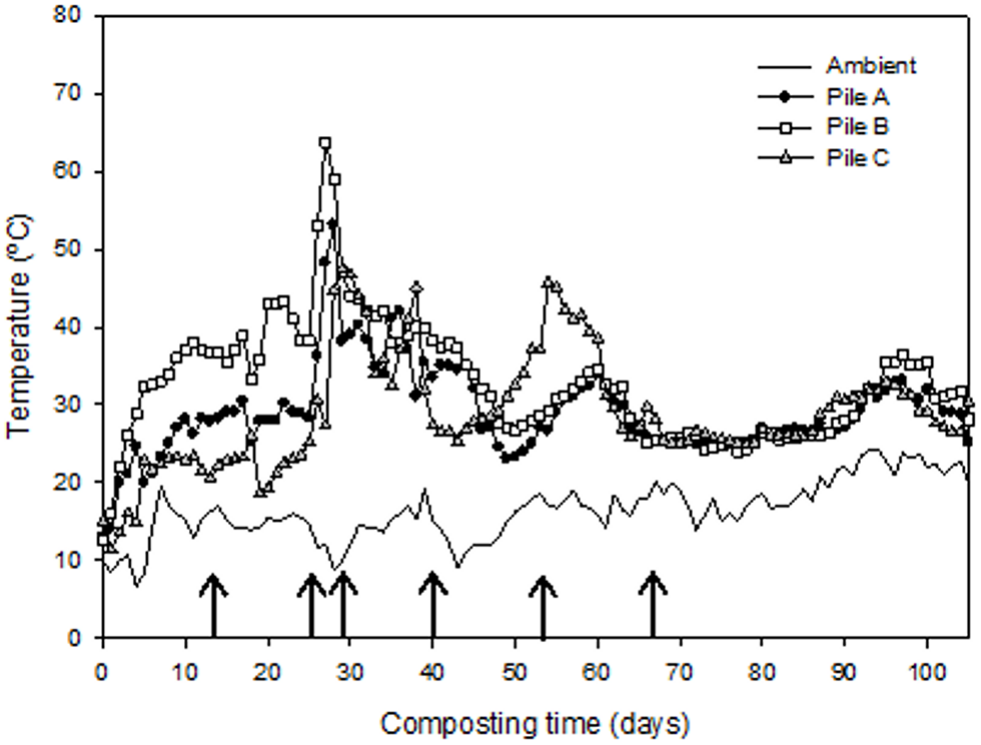
Temperature profiles of the composting piles. Pile A [76% exhausted grape marc + 24% cattle manure]; pile B [72% grape marc + 28% cattle manure]; and pile C [67% exhausted grape marc + 33% poultry manure].

The temperature profile in the composting process determines the rate at which many of the biological reactions take place, being a signalling parameter about nutrient bioavailability and the presence of potential limiting factors (salinity, polyphenols, etc.); also, it is associated to the capacity of the process to reduce the pathogen contents [8]. At the beginning of the process, the temperature increased very slowly in all the mixtures except for pile B (Fig 1), probably as a consequence of its higher contents in water-soluble easy-degradable compounds (Table 2), due to the use of GM, with greater amounts of these compounds than EGM [2]. The initial inhibition of the thermophilic phase during composting of winery and distillery wastes has been reported by other authors [7, 10, 29]. The presence of compounds with antimicrobial effect, such as polyphenols, and the acidic character of this type of wastes could be responsible for this temperature profile [10, 29, 30]. The use of the manures as co-composting ingredients, in general, induced better conditions in the initial composting mixtures, reducing some of the limiting factors linked to winery-distillery wastes, such as the acidic character, since the pH of the initial mixtures (pH = 7.9 for pile A; pH = 7.1 for piles B and C) (S1 Table) was in the range 6–8, suggested as suitable for composting [8]. However, in pile C, the use of PM seemed not to have the same positive effect, showing this pile the lowest thermophilic temperature values at the beginning of the process. This initial inhibition could be due to the higher concentrations of polyphenolic compounds of this waste (Table 1), which induced the highest contents of these compounds in the initial mixture of pile C (2568 mg/kg for pile C compared to 1125 mg/kg for pile A and 1446 mg/kg for pile B) (S1 Table). The turnings carried out throughout composting, especially the second one, reactivated the process in all the mixtures by the increase in the microbial activity, producing the maximum temperature rise in all the composting mixtures. This reactivation of the composting process with turnings was also reported by Bustamante et al. [29] during an experiment of composting of anaerobic digestate using different bulking agents. According to the EXothermic Index, EXI (calculated as the summation of the daily value obtained by subtracting the ambient temperature from the temperature value in the composting pile during the bio-oxidative phase of composting, and expressed as cumulated°C), pile B had the most exothermic behavior (1741 cumulated°C), while piles A and C were quite similar (1344 and 1317, respectively), probably due to the previously commented different content in labile compounds in GM and EGM, the last one obtained after grape marc-washing processes carried out in the distillery.

**Table 2:** Evolution during composting of organic matter-linked parameters usually considered in the classical analytical approach (dry matter basis).

Composting phase ^a^	TOC/TN	WSC (%)	WSC/TN	Cfa (%)	Cha (%)	HR (%)	HI (%)	Pha (%)	Cha/Cfa
*Pile A*: *76% exhausted grape marc + 24% cattle manure*									
I (0)	21.9 ± 0.4	3.14 ± 0.01	1.42 ± 0.04	3.54 ± 0.16	2.98 ± 0.09	12.99 ± 0.23	5.94 ± 0.14	45.72 ± 1.89	0.84 ± 0.06
T (28)	18.4 ± 1.4	2.80 ± 0.03	1.10 ± 0.06	3.14 ± 0.07	3.02 ± 0.04	12.89 ± 0.44	6.32 ± 0.19	49.01 ± 0.20	0.96 ± 0.01
E (105)	17.9 ± 0.8	2.70 ± 0.01	1.05 ± 0.05	2.77 ± 0.05	1.60 ± 0.05	9.33 ± 0.02	3.41 ± 0.12	36.58 ± 1.18	0.58 ± 0.03
M (168)	17.4 ± 0.4	1.10 ± 0.03	0.42 ± 0.02	1.24 ± 0.01	1.16 ± 0.03	5.23 ± 0.01	2.52 ± 0.04	48.15 ± 0.86	0.93 ± 0.03
*Pile B*: *72% grape marc + 28% cattle manure*									
I (0)	21.1 ± 0.0	4.27 ± 0.00	1.82 ± 0.01	3.86 ± 0.05	4.59 ± 0.11	17.24 ± 0.42	9.20 ± 0.27	54.46 ± 0.23	1.20 ± 0.01
T (28)	19.2 ± 0.8	3.72 ± 0.09	1.54 ± 0.11	4.08 ± 0.07	4.61 ± 0.01	18.04 ± 0.02	9.57 ± 0.08	53.06 ± 0.48	1.13 ± 0.02
E (105)	17.6 ± 0.5	3.51 ± 0.00	1.35 ± 0.03	3.13 ± 0.06	4.00 ± 0.08	15.43 ± 0.43	8.65 ± 0.24	56.06 ± 0.01	1.28 ± 0.00
M (168)	17.1 ± 0.0	1.86 ± 0.12	0.69 ± 0.05	2.91 ± 0.01	1.43 ± 0.10	9.44 ± 0.26	3.11 ± 0.23	32.97 ± 1.50	0.49 ± 0.03
*Pile C*: *67% exhausted grape marc + 33% poultry manure*									
I (0)	14.8 ± 0.3	3.29 ± 0.04	1.15 ± 0.01	3.53 ± 0.04	2.69 ± 0.25	17.24 ± 0.69	5.96 ± 0.57	54.46 ± 2.01	1.20 ± 0.06
T (28)	12.7 ± 0.2	2.84 ± 0.02	1.05 ± 0.02	2.95 ± 0.01	3.35 ± 0.06	13.87 ± 0.12	7.37 ± 0.14	53.14 ± 0.54	1.13 ± 0.02
E (105)	13.9 ± 0.3	2.68 ± 0.01	0.85 ± 0.01	3.05 ± 0.10	1.84 ± 0.05	11.09 ± 0.20	4.18 ± 0.08	37.66 ± 1.38	0.60 ± 0.07
M (168)	13.5 ± 0.1	0.98 ± 0.01	0.31 ± 0.00	1.43 ± 0.06	0.81 ± 0.01	5.34 ± 0.17	1.93 ± 0.02	36.20 ± 0.71	0.57 ± 0.02

TOC: total organic C; TN: total organic N; WSC: water-soluble C; Cfa: fulvic acid-like C; Cha: humic acid-like C; HR: humification ratio; HI: humification index; Pha: percentage of humic acid-like C; Cha/Cfa: ratio of humic acid-like C/fulvic acid-like C.

^a^ Days in brackets.

I: initial phase of composting; T: thermophilic phase of composting; E: end of the bio-oxidative phase; M: maturity phase. Values reported as mean ± standard error (n = 3).

### Organic matter evolution during composting: classical analytical approach

The organic matter degradation profile during composting, as determined by OM losses (data not shown), followed a first-order kinetic equation in the composting piles, OM losses (%) = A (1–e^-*k*t^). Curve fitting of the experimental data gave the following parameter values (standard deviation in brackets):

$$\text{Pile A}:\;\text{A}=49.6\;\left(8.1\right),\;k=0.0131\;\left(0.0040\right),\;\text{RMS}=0.878,\;\text{F}=65.8^{***},\;\text{SEE}=4.89$$

$$\text{Pile B}:\;\text{A}=44.2\;\left(6.7\right),\;k=0.0215\;\left(0.0072\right),\;\text{RMS}=0.769,\;\text{F}=31.0^{***},\;\text{SEE}=6.45$$

$$\text{Pile C}:\;\text{A}=50.4\;\left(9.1\right),\;k=0.0091\;\left(0.0030\right),\;\text{RMS}=0.880,\;\text{F}=95.8^{***},\;\text{SEE}=5.99\;,$$

where RMS is the residual mean square. All equations were significant at P < 0.001. The OM degradation kinetics of all the piles fitted satisfactorily this equation. The A and *k* values obtained were lower than those reported by different authors in other composting experiments using livestock and agroindustrial wastes [8, 31, 32], probably due to the composting scale used, with a pile weight much lower than those of the commented experiments. The maximum degradation of OM (A) was observed in pile C, but the greatest OM degradation rate was observed in pile B (in accordance with the highest previously commented EXI), since this pile showed the highest values of *k* and the product of A x *k*, showing the highest degradation rate the mixture with GM compared to those with EGM.

All the piles showed a decrease in the TOC/TN ratio values (Table 2), especially at the beginning of the composting process and in piles A and B, corresponding to the highest OM degradation rates observed in these piles, showing pile C the lowest decrease in the TOC/TN ratio. At the end of the composting process, the TOC/TN ratio reached values <20 in all piles, suggesting that all composts had reached an acceptable degree of maturation [4]. However, in pile C, the initial TOC/TN ratio value was lower than the reference value for mature compost and therefore, this maturity parameter cannot be used as the only maturity indicator for this pile. Thus, this fact evidences the previously commented need of using different parameters to estimate compost maturity.

The water-soluble organic C represents the most active fraction of carbon and is indicative of compost stability, since it is constituted by sugars, hemicellulose, phenolic substances, amino acids, peptides and other easily biodegradable substances [33]. Therefore, the study of the transformations occurring in the soluble OM can be useful for assessing compost maturity. In this sense, the evolution of the water-soluble organic C and of the water-soluble organic C to the total organic N ratio (WSC/TN) can be considered as suitable parameters for assessing compost maturity [4]. The contents of water-soluble C decreased in all the piles throughout the composting process (Table 2), due to the degradation of simple, water-soluble organic compounds [8]. At the end of the process, in general, all the piles had values lower or close to 1.7%, the maximum value suggested for a compost to be considered mature [4]. The WSC/TN also decreased in all the piles throughout the composting process (Table 2), with decreases in relation to the initial value of 70%, 62% and 73% for the piles A, B and C, respectively, due to the degradation of simple water-soluble organic compounds, such as sugars, amino acids, and peptides [33]. The final values of this ratio ranged between 0.31 and 0.69, within the limit value established for a mature compost [4].

Regarding the humification parameters, such as the humification indexes (HI, HR, Pha, Cha/Cfa) and/or the contents in humic and fulvic acid-like C (Cha and Cfa), their increases during composting have been reported in different studies as indicative of OM humification [4]. However, in this experiment, these parameters have not shown the classical trend of increasing throughout the composting process, showing, in general, all the piles a decrease from the initial values (Table 2), this fact being also observed by other authors [8, 34]. This could be attributed to the alkaline co-extraction and partial acid co-precipitation of incompletely or not humified components of organic matter, such as the polyphenols present in the winery and distillery wastes, which hid the real evolution of the humic fraction. This fact was reported by Marhuenda-Egea et al. [18] that confirmed this interference using fluorescence excitation–emission matrix to determine humic-fulvic evolution during composting of winery and distillery residues. Moreover, this shows the high dependence of these parameters on the origin of the raw materials used, being not useful to evaluate the humification in all types of compost, highlighting the need of using other analytical and/or instrumental techniques to evaluate compost maturity.

### Organic matter evolution during composting: advanced instrumental approach

#### Thermal tools

**Fig 2:**
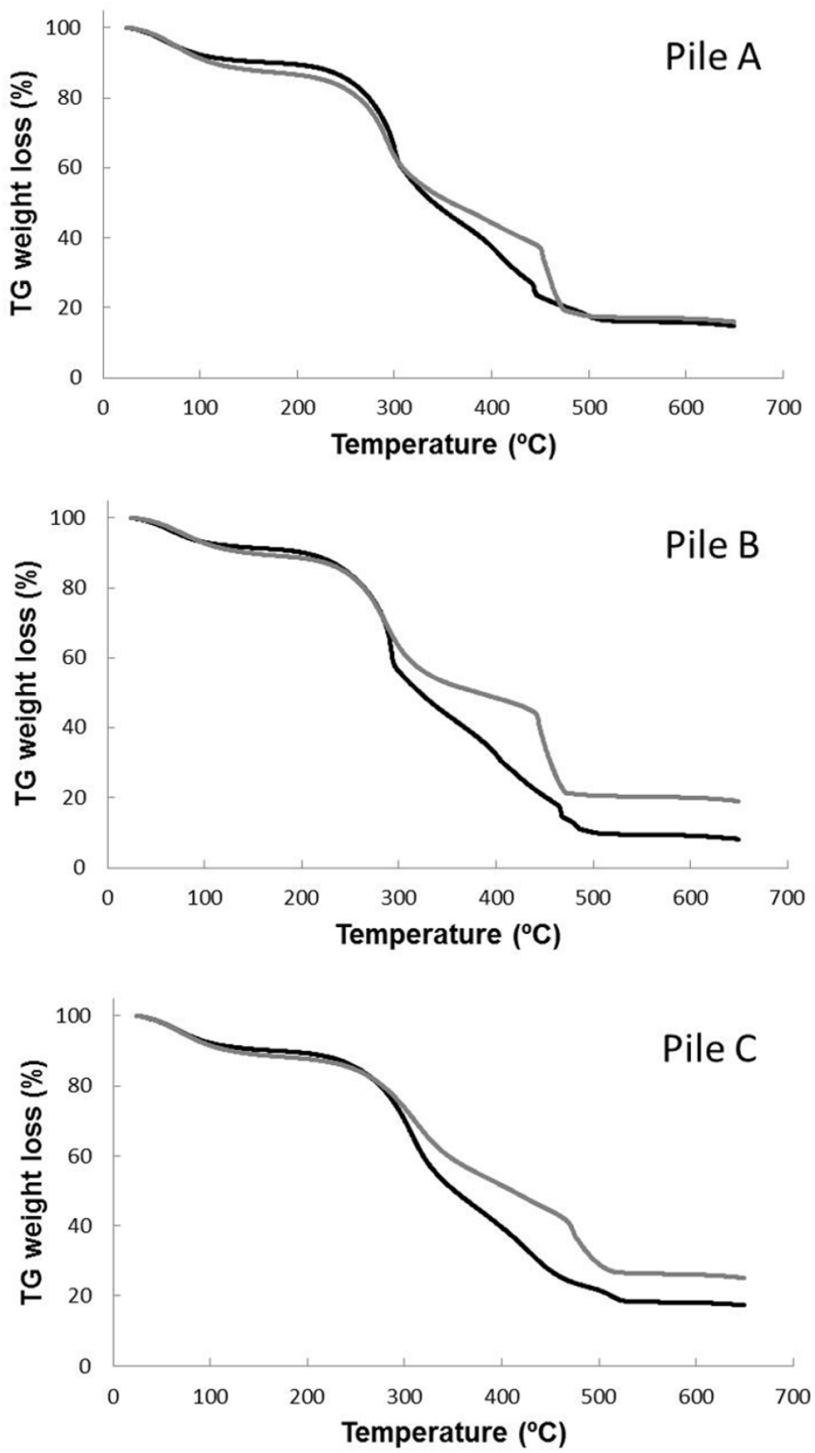
TG curves for the compost samples of piles A, B and C. Black line corresponds to the samples at the initial phase of the composting process and the grey line corresponds to the mature composts.

**Fig 3:**
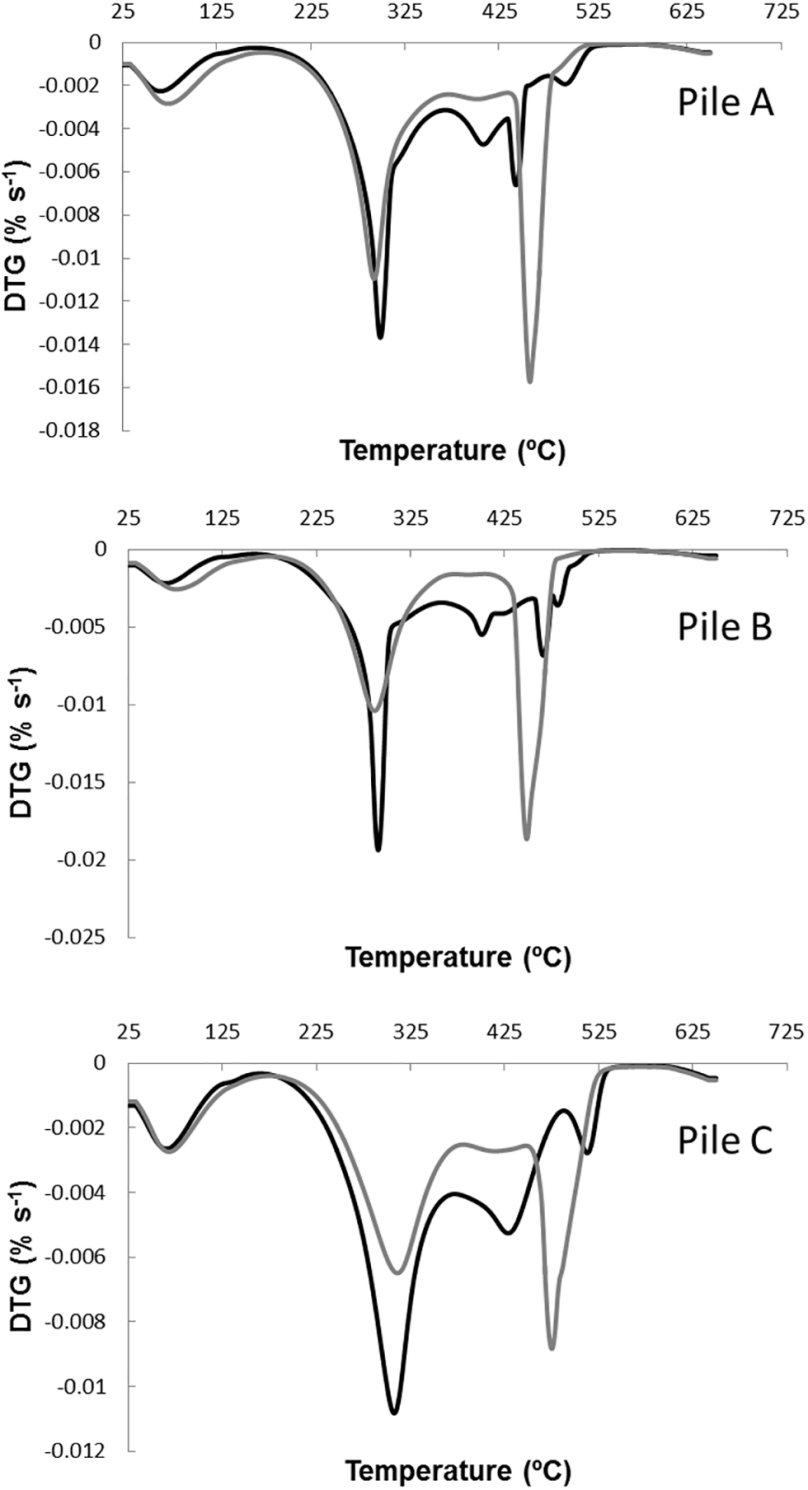
DTG curves for the compost samples of piles A, B and C. Black line corresponds to the samples at the initial phase of the composting process and the grey line corresponds to the mature composts.

Thermogravimetric analysis (TG, DTG and DTA) were carried out to assess the changes in organic matter during the composting process. The thermograms of the compost samples for the piles A, B and C, corresponding to the initial and maturity stage of composting are shown in Fig 2. These thermograms displayed different steps-regions in the thermal analysis, linked to the complexity of the organic matter present in the compost samples. In the presence of atmospheric oxygen, two exothermic phenomena may occur in compost characterization, such as volatilization of aliphatic compounds or carbohydrates and the oxidation of high molecular weight compounds [35]. A clear change within the range of 250°C to 550°C is shown, which corresponds to the combustion of carbohydrates, aromatic compounds and other substances [35]. The three piles showed a similar behavior, the amount of matter that was burned being higher in the initial sample than in the corresponding mature compost sample at the same temperature. This trend suggests a progressive transformation of the biomass in the polyelectrolyte macromolecules known as humified matter and thus, the increase in the molecular weight, stability, and aromatization degree during the co-composting process [19, 36]. The heat-labile material was decomposed in the early stages of composting, producing that with time the most recalcitrant material become the material predominant in the compost piles. In the DTG profiles, three peaks can be distinguished between 250 and 530°C (Fig 3), associated to the degradation of organic matter [35]. The first peaks were obtained in the range between 250 and 400°C and the third one appeared between 450 and 500°C. The two peaks within the range of 250–420°C could be attributed to the combustion of carbohydrates, such as cellulose and lignocellulosic [35], which are main components of the plant material present in the winery and distillery wastes. The first peak decreased considerably with time in the piles B and C; however, in the samples of pile A only a slight change was observed (Fig 3). This fact could be due to a higher availability of easily degradable compounds, such as carbohydrates and proteins, in these piles. This confirms the previous results concerning the higher degradation rate observed in these piles, especially in pile B, using the classical analytical approach. Moreover, the contents in carbohydrates in the piles decreased throughout the composting process due to the microbial degradation processes, implying an enrichment in recalcitrant material due to the concentration effect. These recalcitrant compounds constitute the fraction of material that combusted in the range of 450 and 500°C. Different authors [35, 37] have also attributed the range between 350–500°C to the degradation of complex aromatic structures, such as the humified organic matter. Therefore, more stabilized samples take more energy for decomposition, e.g. require higher temperatures to achieve the same mass losses, due to these samples are richer in highly complex aromatic compounds compared to the initial ones, which indicates the OM stabilization during the composting process.

**Fig 4:**
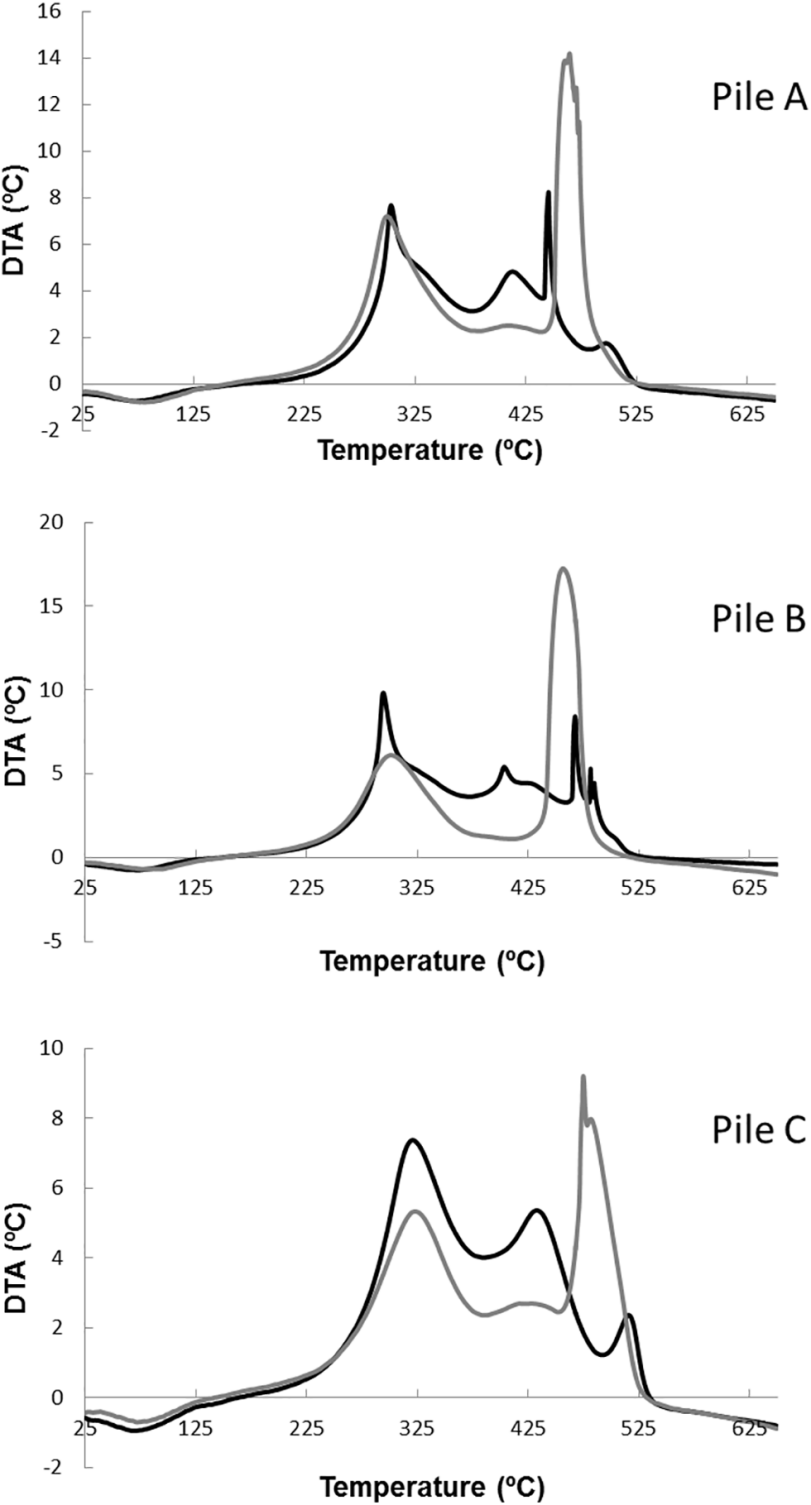
DTA curves for the compost samples of piles A, B and C. Black line corresponds to the samples at the initial phase of the composting process and the grey line corresponds to the mature composts.

The DTA profiles of the organic matter displayed two distinct exothermic peaks (between 250–400°C and between 400–580°C) (Fig 4), which indicated the thermal reactions of organic components characterized by different thermal stability [19, 37]. A decrease in the relative intensity of the first exothermic peak was observed in DTA profiles. This fact indicates the progressive degradation of the carbohydrates and aliphatic compounds [19, 38] and of some easily biodegradable aromatic structures [39]. At the same time, an increase in the second peak intensity was observed in sequential samplings of the composting piles. This peak is associated to more complex aromatic structures with high molecular weights, such as lignin [39, 40]. The increase in the intensity of this peak can be related to the release of the aromatic structures after deterioration of the lignocellulose complex and thus, the condensation of these structures [40]. Therefore, this peak can be attributed to the increase in the aromatic fraction in the mature samples. These exothermic reactions were associated with the peaks in the TG and DTG curves, linked with loss weight, but not with energy. The indexes R1 and R2 can summarize the observed organic matter transformations [41]. R1 is the ratio between the mass loss associated with the two main exothermic reactions at TG (Table 3), and R2 is the ratio of the peak areas in the DTA analysis. In all the piles, the R1 ratio increased during composting, thus revealing a high sensitivity of this parameter to the chemical changes induced by the bio-transformation of organic materials. This shows the relative amount of the most thermally-stable fraction of the organic matter with respect to the less stable one. R2 behavior was similar to the R1, but the difference associated between the initial and mature samples was higher than in R1. R2 evolution showed that the most labile organic matter (i.e., cellulose), which requires less energy for its combustion, disappeared during the first stages of the composting process, remaining an organic matter more complex and therefore, with higher thermal stability (Table 3).

**Table 3:** R1 and R2 indexes from TG and DTA analysis of the compost samples at the initial (I) and maturity (M) phases of composting.

	TG (% mass losses)			DTA (peak area)		
	Peak 1 (P1)	Peak 2 (P2)	R1 (P2/P1)	Peak 1 (P1)	Peak 2 (P2)	R2 (P2/P1)
*Pile A*: *76% exhausted grape marc + 24% cattle manure*						
I	51.9	19.8	0.38	572	76	0.13
M	42.2	26.7	0.63	576	902	1.57
*Pile B*: *72% grape marc + 28% cattle manure*						
I	57.6	22.3	0.39	752	137	0.18
M	39.9	27.8	0.70	891	1425	1.60
*Pile C*: *67% exhausted grape marc + 33% poultry manure*						
I	49.6	18.0	0.36	804	114	0.14
M	36.0	22.5	0.62	699	696	1.00

These results suggested an enhancement of the number of stable compounds through two ways, either by a concentration effect due to the loss of the most labile material during composting and/or by the novo synthesis of more stable and complex compounds, such as the humic-like substances. Presumably, both processes occur simultaneously due to the microbial activity during the composting process.

#### Spectroscopic tools: FT-IR and CP-MAS ^13^C-NMR techniques

**Fig 5:**
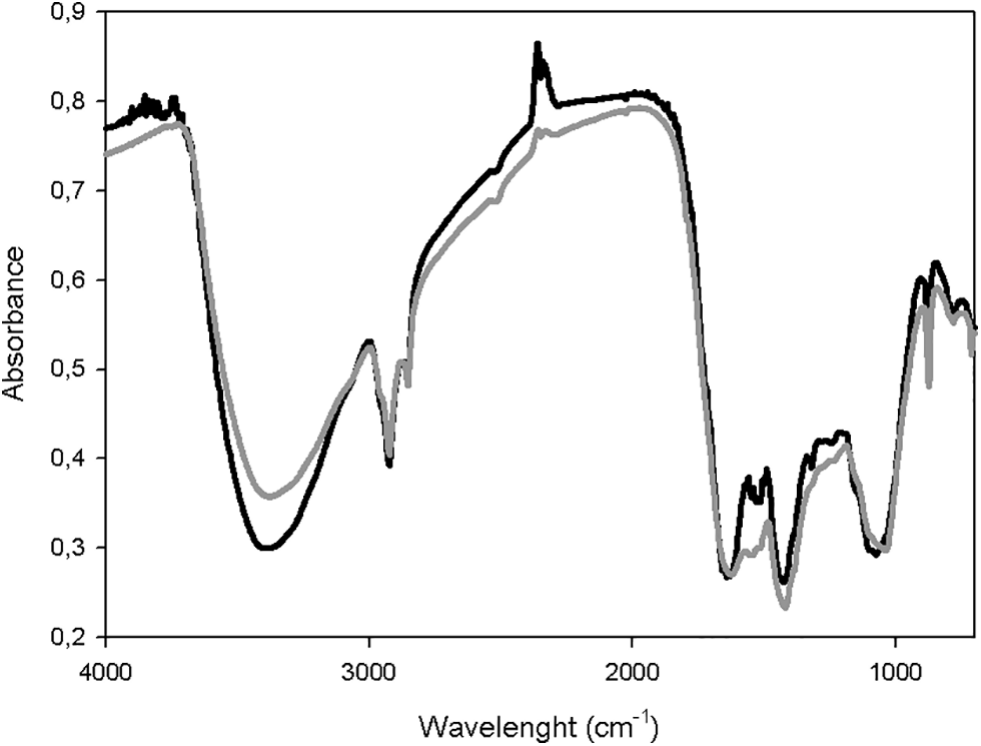
FTIR spectra of the samples of pile C during the composting process. Initial stage (black line) and maturity stage (grey line).

FT-IR spectra from the most representative samples (initial and mature samples of pile C) are displayed in Fig 5. Some of the most significant peaks are pointed. The variations in the FT-IR spectra during composting time were minor. The region between 2930–2880 cm^-1^ could be attributed to aliphatic groups in fatty acids [22] and its stationary behavior during the composting indicated the presence of vegetable material highly resistant to microbiological degradation, probably associated to molecules of vegetable origin like cutines, suberines or lignins [18]. Variations between piles and time of sampling in the FT-IR spectra were appreciated between 1800 and 600 cm^-1^ region. The band around 1540 cm^-1^ can be assigned to amide II and components containing lignin. These bands were identified in biowastes due to their content of wood and plants, which are rich in lignin [20]. The peak at 1420 cm^-1^ was due to the OH in-plane bend of carboxylic acids, the CO_2_ stretch of carboxylates and the aliphatic CH_2_ group of alkanes. The band at 1384 cm^−1^ was assigned to nitrate and inorganic components as carbonates absorb at 875 cm^-1^. However, a visual inspection of the FT-IR spectra bands of the samples did not show valuable differences among the samples during the composting process. In order to detect possible variations of the FT-IR bands during composting, the relative absorbance (rA) of certain signals was used [11, 20]. The relative absorbance is the height of one distinct band multiplied by 100 and divided by the sum of all compared band heights [42]. Bands heights were measured and corrected referring to the chosen baseline by OMNIC 5.1b software. Eight bands were used to calculate relative absorbances (rA): 2927, 2854, 1640, 1548, 1428, 1384, 1037, and 875 cm^-1^ (Table 4).

**Table 4:** Relative absorbance in % of the sum of all the peak heights of the FT-IR spectra of the composting samples.

	Relative absorbance (%)								
Composting phaseª	2927 cm^-1^	2854 m^-1^	1640 cm^-1^	1548 cm^-1^	1420 cm^-1^	1384 cm^-1^	1037 cm^-1^	875 cm^-1^	1037/1384 ratio
*Pile A*: *76% exhausted grape marc + 24% cattle manure*									
I (0)	10.4	12.9	9.9	12.6	10.7	12.5	11.4	19.6	0.91
T (28)	12.7	15.5	8.8	11.3	11.5	12.0	9.8	18.5	0.81
E (105)	13.2	16.3	8.5	10.5	11.0	11.4	10.2	19.0	0.89
M (168)	13.8	16.3	8.9	10.2	10.8	11.3	10.4	18.2	0.92
*Pile B*: *72% grape marc + 28% cattle manure*									
I (0)	11.8	14.7	9.3	12.0	11.9	12.2	10.0	18.2	0.82
T (28)	12.4	15.2	9.2	11.5	11.6	12.0	10.3	17.9	0.86
E (105)	12.4	14.7	10.1	11.5	11.8	11.9	10.7	17.1	0.89
M (168)	11.9	15.2	9.5	10.2	10.7	11.8	11.3	19.3	0.96
*Pile C*: *67% exhausted grape marc + 33% poultry manure*									
I (0)	13.6	16.7	9.2	12.3	9.1	10.9	10.8	17.5	0.99
T (28)	13.4	15.7	10.1	12.3	9.9	11.1	11.3	16.2	1.02
E (105)	13.6	15.9	10.5	12.1	9.5	10.6	11.2	16.6	1.06
M (168)	14.9	17.6	10.0	10.6	8.5	10.0	10.8	17.5	1.08

^a^ Days in brackets.

I: initial phase of composting; T: thermophilic phase of composting; E: end of the bio-oxidative phase; M: maturity phase.

In this experiment, only the rA ratio between 1037 and 1384 cm^-1^ signal showed changes with time and type of pile, associated to the infrared (IR) spectra bands assigned to the C/N ratio (1037 and 1384 cm^-1^). Piles B and C showed increases of 17 and 9% in this ratio during the composting that were negatively correlated to the observed increase of the TOC/TN ratio using the classical analytical approach, which could be used as indicator of compost stability. In addition, there was an increase in the values of rA of signals attributed to C aliphatic (2927 cm^-1^ band); however, the latest aspect was only clear for pile C. These results could be explained by the fact that these signals are generated by molecules of vegetable origin, very resistant to degradation, which could induce a concentration effect, since other labile compounds are degraded during the composting process.

**Fig 6:**
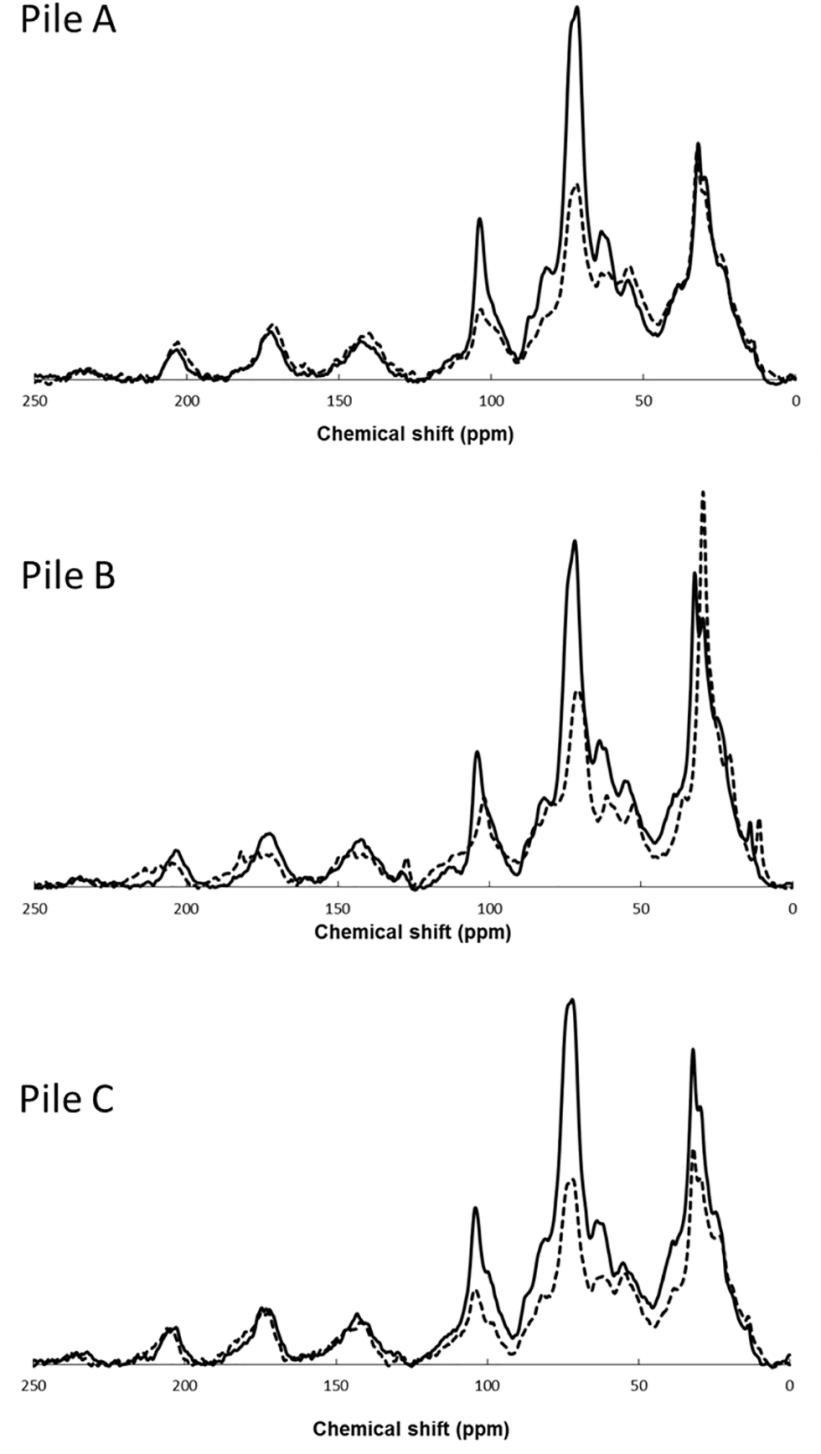
CPMAS ^13^C-NMR spectra of the samples of piles A, B and C during composting. Beginning (solid line) and end (dotted line).

The CP-MAS ^13^C-NMR spectra show several principal peaks corresponding to the samples collected at the beginning of the composting process at 204, 175, 142, 130, 105, 72, 62, 55, 33, 30 and 24 ppm (Fig 6). An increase of alkyl C in the 0–50 ppm region, associated to the degradation of aliphatic components was measured for the sample of pile B. This result could be due to the breakdown of polysaccharides, presumably in higher amount in pile B due to the use of GM as component, yielding simple alkyl chains [21, 23]. A shoulder appears around 25 ppm in CP-MAS ^13^C-NMR spectra, indicating the presence of methyl groups in alkyl chains. The methyl group presents an elevated mobility and, therefore, a weak coupling [11]. At 30 and 33 ppm, appear the methyl and methylen groups, respectively. The main difference between methyl and methylene groups is related to carbon dipolar interactions with linked protons. The peak that appears around 55 ppm in CP-MAS ^13^C-NMR spectra supports the assignment to Cα of polypeptides [43]. However, the signal at 55 ppm in CP-MAS ^13^C-NMR spectra can be also assigned to O-CH3 groups in lignin (phenolmethoxyl of coniferyl and sinapyl moieties) and in hemicellulose (glucoronic acid in xylan) [19]. These composts principally derived from plant and organic remains, and thus, contain different biomolecules, such as proteins and peptides, as well as lignin and hemicellulose. So, the signal around 55 ppm has contribution from Cα of polypeptides and O-CH3 groups in lignin and in hemicellulose [44]. Also, the spectra were dominated by a strong signal around 72 ppm, assigned to cellulose and hemicellulose in CP-MAS spectra. The chemical shift at 105 ppm has been assigned to different carbons of lignin-type moieties in CP-MAS spectra: The C2 carbons of both guaiacyl and syringyl lignin structures and the C6 carbon of syringyl units. This peak could be also attributed to quaternary aromatic carbons in tannins [11]. However, the low signal to noise (S/N) ratio in these spectra made difficult the assignment. In the CPMAS ^13^C-NMR spectra, the region between 110 ppm and 170 ppm can be divided in two sub-regions: the first one, between 110–140 ppm is assigned to non-substituted aromatic C and C-substituted aromatic carbons [45]. The second region, between 140–160 ppm is attributed to aromatic carbons linked to O or N. The first region only shows peaks at 112 and 130 ppm. The second sub-region, between 140–160 ppm, exhibits peaks usually attributed to lignin or tannins [11]. The peak centered at 142 ppm is attributed to methoxy-substituted or hydroxy-substituted phenyl C [11]. The peak at 153 ppm is assigned to oxygen-substituted aromatic C, including both C-OCH3 and C-OH groups [46]. Also, the region between 170–210 ppm is assigned to carbonyl/carboxyl carbons of ester and amide groups [46]. The contribution of amides at the signals in this region should be important, because the nitrogen content is high (Table 5).

**Table 5:** Relative intensity (%) of each C type by integration of the solid-state ^13^C NMR spectra of the compost samples. The chemical shift regions were: A) alkyl (45 to -10 ppm); B) N-alkyl/methoxy (60–45 ppm); C) O-alkyl (95–60 ppm); D) O2-alkyl (110–95 ppm); E) aromatic (145–110 ppm); F) O-aromatic (165–145 ppm); G) carbonyl (210–165 ppm).

	Chemical shift regions							
Composting phaseª	A	B	C	D	E	F	G	Alkyl/O-Alkyl ratio
*Pile A*: *76% exhausted grape marc + 24% cattle manure*								
I (0)	30.7	17.0	31.7	7.8	3.3	3.5	6.1	0.97
T (28)	36.4	15.9	23.2	8.0	5.7	3.7	7.3	1.60
E (105)	37.9	16.1	21.2	7.7	6.3	3.9	6.9	1.79
M (168)	37.3	14.3	15.5	6.9	7.1	4.8	14.2	2.40
*Pile B*: *72% grape marc + 28% cattle manure*								
I (0)	33.6	15.3	28.6	8.5	2.9	3.7	7.4	1.18
T (28)	34.3	13.7	28.1	9.2	3.6	3.6	7.5	1.22
E (105)	33.3	14.8	29.1	9.5	3.8	3.0	6.6	1.14
M (168)	34.9	15.2	25.9	8.5	3.3	4.0	8.2	1.35
*Pile C*: *67% exhausted grape marc + 33% poultry manure*								
I (0)	30.8	14.2	30.5	10.5	3.5	3.5	7.0	1.01
T (28)	34.4	14.6	26.9	8.5	3.2	4.0	8.5	1.28
E (105)	33.9	15.5	26.8	8.9	3.7	3.9	7.5	1.27
M (168)	34.3	15.5	23.6	7.9	3.4	5.0	10.2	1.45

^a^ Days in brackets.

I: initial phase of composting; T: thermophilic phase of composting; E: end of the bio-oxidative phase; M: maturity phase.

The area under the curves in the CP-MAS ^13^C-NMR spectra was calculated for the different regions (the regions are detailed in Materials and Methods section) (Table 5). The main C types are O-alkyl (95–60 ppm) and alkyl (45 to -10 ppm). These two regions experimented opposite evolutions with time. The O-alkyl (from cellulose and hemicellulose) signals decreases with the composting time, especially in piles A and C (Fig 6 and Table 5). Moreover, in all the piles, the intensity of the alkyl signals (from aliphatic chains, such as lipids, cutin or suberin) increases in the mature composts. Aliphatic structures of cutin and suberin molecules are resistant to biodegradation and thus, these molecules could have been accumulated throughout the composting process [36]. This shows a concentration effect over the C-alkyl by a preferential degradation of sugar polymer, as cellulose and hemicellulose, during the composting process (Fig 6) [36]. This preferential degradation pathway of cellulose and hemicellulose observed in the CP-MAS ^13^C-NMR data agreed with the previous thermal analysis data. Minor components in the CP-MAS ^13^C-NMR spectra of the compost samples were carbonyl (210–165 ppm), O-aromatic (165–145 ppm), aromatic (145–110 ppm), O2-alkyl (110–95 ppm), and N-alkyl/methoxy (60–45 ppm) [37]. The scatter of the data (Fig 6) could reflect the non-quantitative characteristic of the CP-MAS ^13^C-NMR technique, as the errors show (the observable carbon contents for this type of compost samples were generally around 60–66%).

### Quality of the composts obtained

The main physico-chemical, chemical and biological properties of the final composts obtained are shown in Table 6. The composts obtained had final pH values close to neutrality and within the range (6.0–8.5) suggested as suitable for compost [47]. The EC values in all the composts were close to 2 dS/m, probably due to the presence of manure in the formulation of all the piles, showing the composts elaborated with CM (A and B), the greatest EC values. However, these values were similar or lower to those reported in composts with similar origin [7, 8, 10].

**Table 6:** Main properties of the mature composts obtained (dry matter basis).

Parameter	Compost A	Compost B	Compost C
pH	7.37 ± 0.01	7.98 ± 0.01	7.62 ± 0.02
EC (dS m^-1^)	2.19 ± 0.05	2.88 ± 0.02	1.57 ± 0.02
OM (%)	80.7 ± 0.9	83.6 ± 0.3	76.7 ± 0.3
TN (g kg^-1^)	2.64 ± 0.04	2.69 ± 0.01	3.22 ± 0.01
P (g kg^-1^)	5.5 ± 0.2	4.7 ± 0.1	9.0 ± 0.4
Na (g kg^-1^)	6.1 ± 0.0	4.7 ± 0.2	1.8 ± 0.1
K (g kg^-1^)	18.9 ± 0.6	27.8 ± 0.1	18.8 ± 0.3
Ca (g kg^-1^)	39.7 ± 0.4	33.5 ± 2.1	66.0 ± 2.8
Mg (g kg^-1^)	4.6 ± 0.5	4.7 ± 0.3	4.3 ± 0.3
Fe (mg kg^-1^)	1214 ± 46	1364 ± 111	984 ± 53
Mn (mg kg^-1^)	120 ± 1	101 ± 9	173 ± 2
Cu (mg kg^-1^)	32.8 ± 0.3	34.4 ± 0.1	58.5 ± 0.6
Zn (mg kg^-1^)	160 ± 4	132 ± 2	165 ± 5
WSPOL (mg kg^-1^)	552 ± 2	982 ± 8	432 ± 29
GI (%)	78.2 ± 1.9	68.4 ± 1.4	61.8 ± 0.3

EC: electrical conductivity; OM: organic matter; TN: total organic nitrogen; WSPOL: water-soluble polyphenols; GI: germination index. Values reported as mean ± standard error (n = 3).

Organic matter (OM) concentrations were similar in all the composts and higher than the minimum values established by the Spanish and the European legislation (35% and 30%, respectively for OM) [48, 49]. TN contents were greater than 2% in all the composts, which were affected by the manure nature, showing the compost elaborated with PM (compost C) the highest TN concentrations. These contents were similar to those reported in composts elaborated using agroindustrial wastes and manures [8, 10, 12, 28, 29, 32]. The concentrations in other macronutrients, such as P and K, were higher or similar to those found in other materials usually considered as organic amendments, such as composts from agroindustrial wastes and manures [12] K contents in the compost B, elaborated using GM, were significantly higher than in the rest, due to the higher contents of water-soluble elements in this material [2], while P contents were similar in the composts A and B, elaborated using CM, and lower than in compost C, due to the use of PM. Contents of Ca ranged between 34–66 g/kg, showing compost C higher levels, and Na contents were significantly lower than those reported for manure-derived composts [29], this aspect being an additional added value of these composts. The micronutrient concentrations were similar or lower than those observed in composts from agroindustrial wastes and manures, previously mentioned, the values of Cu and Zn being lower than the limit values established for compost by the Spanish legislation and the European guidelines [48, 49].

Regarding other parameters related to the compost maturity and/or stability, the contents in water-soluble polyphenols, compounds that in high levels inhibit the germination [8], were quite lower than those observed in the raw materials. All the composts also showed absence of phytotoxicity, with values of the germination index > 50%, the minimum value established by Zucconi et al. [24].

## Conclusions

In conclusion, the classical analytical methods have shown a suitable development of the composting process in all the piles, with a more significant effect of the winery-distillery waste used than of the type of manure, but obtaining in all the scenarios end-products with suitable properties and a good degree of maturity for their agricultural use. However, the classical analytical approach has also been shown to be ineffective to study the humification process during composting of this type of materials. Therefore, thermal analysis techniques has been useful to elucidate the degradability of the remaining material and additionally, to assess qualitatively the rate of OM stabilization and recalcitrant C in the compost samples; FT-IR has allowed to identify functional groups in composting, which indicates composted material stability and maturity; while CPMAS ^13^C-NMR has provided semi-quantitatively partition of C compounds and structures during the process, being especially interesting their variation to evaluate the biotransformation of each C pool, especially in the comparison of recalcitrant C vs labile C pools, e.g. Alkyl /O-Alkyl ratio. Therefore, the combination of instrumental techniques clearly complemented the existing classical analytical tools, especially those based on non-specific chemical determinations. Also, the knowledge of the structural and functional nature of mature compost will help to predict the behavior of compost in agronomic uses.

## Supporting Information

S1 Fig FTIR spectra of the samples of piles A and B. (TIF)

S1 Table Evolution physico-chemical and other chemical parameters during composting. (DOCX)
