# Supplementary material for: Chemical, Thermal and Spectroscopic Methods to Assess Biodegradation of Winery-Distillery Wastes during Composting
Source: PLoS One. 2015 Sep 29;10(9):e0138925. doi: 10.1371/journal.pone.0138925 (PMC4587889; doi:10.1371/journal.pone.0138925)
Supplement: S1 Table — (DOCX) [file pone.0138925.s002.docx]

Table 7. Evolution physico-chemical and other chemical parameters during composting (dry matter basis).

| Composting phase ^a^ | pH | EC (dS m^-1^) | OM (%) | TOC (%) | WSC (%) | WSPOL (mg kg^-1^) | TN (%) |
| --- | --- | --- | --- | --- | --- | --- | --- |
| *Pile A: 76% exhausted grape marc + 24% cattle manure* | | | | | | | |
| I (0) | 7.87 ± 0.01 | 3.68 ± 0.03 | 88.3 ± 0.7 | 50.2 ± 0.4 | 3.14 ± 0.01 | 1125 ± 210 | 2.29 ± 0.05 |
| T (28) | 7.42 ± 0.08 | 2.29 ± 0.03 | 86.9 ± 0.4 | 47.8 ± 0.8 | 2.80 ± 0.03 | 845 ± 28 | 2.60 ± 0.16 |
| E (105) | 7.22 ± 0.01 | 2.99 ± 0.01 | 81.6 ± 1.8 | 46.8 ± 0.1 | 2.70 ± 0.01 | 744 ± 14 | 2.61 ± 0.12 |
| M (168) | 7.37 ± 0.01 | 2.19 ± 0.05 | 80.7 ± 0.9 | 45.9 ± 0.4 | 1.10 ± 0.03 | 552 ± 2 | 2.64 ± 0.04 |
| *Pile B: 72% grape marc + 28% cattle manure* | | | | | | | |
| I (0) | 7.06 ± 0.02 | 3.30 ± 0.00 | 90.1 ± 0.2 | 49.9 ± 0.3 | 4.27 ± 0.00 | 1446 ± 14 | 2.36 ± 0.02 |
| T (28) | 7.76 ± 0.08 | 2.40 ± 0.06 | 88.2 ± 0.4 | 48.2 ± 0.3 | 3.72 ± 0.01 | 1109 ± 23 | 2.51 ± 0.11 |
| E (105) | 7.51 ± 0.01 | 3.49 ± 0.02 | 83.7 ± 0.3 | 46.2 ± 2.2 | 3.51 ± 0.00 | 1143 ± 19 | 2.63 ± 0.05 |
| M (168) | 7.98 ± 0.01 | 2.88 ± 0.02 | 83.6 ± 0.3 | 45.9 ± 0.1 | 1.86 ± 0.12 | 982 ± 9 | 2.69 ± 0.01 |
| *Pile C: 67% exhausted grape marc + 33% poultry manure* | | | | | | | |
| I (0) | 7.09 ± 0.06 | 3.00 ± 0.08 | 83.0 ± 0.4 | 45.2 ± 0.1 | 3.29 ± 0.04 | 2568 ± 21 | 3.06 ± 0.06 |
| T (28) | 7.44 ± 0.05 | 2.52 ± 0.01 | 82.0 ± 0.0 | 45.5 ± 0.1 | 2.84 ± 0.02 | 1082 ± 13 | 3.57 ± 0.06 |
| E (105) | 7.22 ± 0.06 | 2.08 ± 0.04 | 77.3 ± 0.1 | 44.1 ± 0.3 | 2.68 ± 0.01 | 656 ± 4 | 3.18 ± 0.04 |
| M (168) | 7.62 ± 0.02 | 1.57 ± 0.02 | 76.7 ± 0.3 | 43.3 ± 0.1 | 0.98 ± 0.01 | 432 ± 29 | 3.22 ± 0.01 |

EC: electrical conductivity; OM: total organic matter; TOC: total organic C; WSC: water-soluble C; WSPOL; water-soluble polyphenols; TN: total organic N.

^a^ Days in brackets. I: initial phase of composting; T: thermophilic phase of composting; E: end of the bio-oxidative phase; M: maturity phase. Values reported as mean ± standard error (n =3).
